# Phytotoxic Effects of Textile Wastewater Contaminants on *Lepidium sativum*: Impact of Ozone-Based Treatment

**DOI:** 10.3390/molecules31214017

**Published:** 2026-11-07

**Authors:** Magdalena Bilińska, Aleksandra Kędzierska-Sar, Ryszard Kapica, Marta Gmurek, Lucyna Bilińska

**Affiliations:** 1 Faculty of Process and Environmental Engineering, Lodz University of Technology, Zeromskiego 116 St., 90-924 Lodz, Poland; magdalena.bilinska@p.lodz.pl (M.B.); 2 Institute of General and Ecological Chemistry, Faculty of Chemistry, Lodz University of Technology, Zeromskiego 116 St., 90-924 Lodz, Poland; aleksandra.kedzierska-sar@p.lodz.pl (A.K.-S.); marta.gmurek@p.lodz.pl (M.G.); 3 Division of Molecular Engineering, Faculty of Process and Environmental Engineering, Lodz University of Technology, Zeromskiego 116 St., 90-924 Lodz, Poland; ryszard.kapica@p.lodz.pl

**Keywords:** phytotoxicity, *Lepidium sativum*, catalytic ozonation, textile wastewater, aniline, naphthol derivative

## Abstract

This study investigates the phytotoxicity of textile wastewater and its degradation by-products using *Lepidium sativum* as a sensitive bioindicator. Model solutions of Reactive Black 5 (RB5), 4-chloroaniline, and H-acid, as well as real textile wastewater containing dye mixtures, were subjected to catalytic ozonation with plasma-deposited thin-film catalysts (based on Co_3_O_4_, Fe_2_O_3_ and W-compounds) and structured substrate (Al_2_O_3_) obtained by thermal treatment of kantal mesh. Phytotoxicity was assessed via the Germination Index (GI), integrating seed germination and root elongation. The degree of pollutant degradation was monitored through absorbance reduction at the characteristic absorption maximum (λmax) and chemical oxygen demand (COD) removal. Catalytic ozonation of RB5 led to 96–97% absorbance reduction and 29–44% COD removal after 45 min and a corresponding GI improvement from 50% to 65%, indicating partial detoxification. However, despite substantial absorbance reductions (91–92% for 4-chloroaniline and 48–75% for H-acid) and COD removal (22–45% and 54–62%, respectively), GI values decreased further, revealing the persistence of toxic intermediates. Real wastewater samples remained highly phytotoxic (GI = 0%), regardless of absorbance and COD decline, likely due to complex composition of the textile effluent matrix with the effect of elevated pH. These findings demonstrate that color or COD alone is insufficient to predict ecological safety, and underscore the need for integrating phytotoxicity assays in evaluating advanced oxidation processes. *Lepidium sativum* proved to be a valuable tool for detecting residual toxicity, supporting its application in environmentally relevant wastewater assessment.

## 1. Introduction

Textile wastewater contains a complex mixture of contaminants that pose significant environmental and health risks. These pollutants include dyes, some metals (like copper, chrome, cobalt, nickel) surfactants, salts, and a variety of organic compounds, many of which are resistant to biodegradation [1,2,3]. Synthetic dyes, in particular, are known for their stability and low degradability, often persisting in water bodies and blocking sunlight penetration, which disrupts aquatic ecosystems [4]. Moreover, the presence of dye-originated toxic substances can inhibit the growth of plants and microorganisms, affect soil quality, and contaminate groundwater when recycled for irrigation [4,5,6,7].

Although the presence of above-mentioned primary pollutants in textile wastewater—such as dyes, heavy metals, and surfactants—has been extensively studied [2], secondary contaminants are not often under scientific interest, despite their potentially greater toxicity. These secondary compounds often originate as transformation products formed during chemical or biological treatment processes, particularly through the degradation of complex dye structures [8,9,10]. Notably, such by-products include various low-molecular-weight organic compounds, including aromatic amines and other intermediates, many of which exhibit high levels of toxicity, persistence in the environment, and bioaccumulative potential [10]. The formation of these secondary pollutants, often more hazardous than the parent compounds, poses significant challenges for ecotoxicological assessment and highlights the necessity for advanced analytical approaches and treatment technologies capable of mitigating both primary and secondary toxicants in textile effluents [7].

Azo dyes, characterized by the presence of one or more azo (-N=N-) chromophores, represent the most widely used class of dyes in the textile industry, accounting for approximately 60–70% of all synthetic dyes produced globally amounting to over 450,000 tons annually [11,12]. These dyes are favored due to their structural versatility, vibrant color range, and relative cost-effectiveness. However, during dyeing processes and subsequent degradation, particularly under reductive or anaerobic conditions, azo dyes can undergo cleavage of the azo bond, resulting in the formation of aromatic amines [11,12,13]. Numerous studies have identified a range of such degradation products in textile wastewater, including aniline, benzidine, o-toluidine, 4-aminobiphenyl and naphthalic acids [12,13,14]. These compounds are of particular concern due to their recognized mutagenic, carcinogenic, and endocrine-disrupting properties [12].

Aniline and its derivatives are among the most concerning aromatic amines found in textile wastewater due to their widespread use and toxicological profiles. Aniline itself is a known toxicant, exhibiting acute and chronic effects in various organisms [15,16]. It has been shown to cause hemolytic anemia, neurotoxicity, and methemoglobinemia in mammals including humans [17,18,19], and its phytotoxic potential has been documented through inhibition of seed germination and root elongation in species such as *Lepidium sativum* [20], Triticum aestivum [6,21], Brassica rapa [22] and Allium cepa [13]. The Effective Concentration 50% (EC_50_) values for aniline’s phytotoxicity are typically in the low mg/L range (203.5 mg/L), indicating significant toxicity even at relatively low concentrations, and some of its derivatives—such as 4-chloroaniline—are considered even more problematic, exhibiting much lower EC_50_ values (e.g., 39.4 mg/L), which reflects their higher phytotoxic potential [22]. Environmental monitoring studies have reported the presence of aniline in surface waters at concentrations ranging from 0.1 to 70 µg/L near industrial discharge points [23]. However, levels can be considerably higher in untreated textile effluents.

Naphthalene derivatives, alongside aniline compounds, are among the most persistent and troublesome environmental pollutants resulting from the breakdown of synthetic dyes. Their presence is due to the fact that many dye molecules contain naphthalene structures as essential components [14]. During the degradation of these dyes, naphthalene derivatives are not fully decomposed and can persist in the environment for extended periods, contributing to long-term contamination [24].

Naphthalene derivatives are not commonly studied as degradation products of synthetic dyes, despite their frequent presence in dye structures. Some scientific studies address their decontamination, focusing primarily on oxidation-based methods such as advanced oxidation processes (AOPs) [25]. However, the toxicity of these degradation products is rarely investigated [26], which limits a full understanding of their environmental and health impacts.

Toxicity studies in the work by Krugly et al. (2015) revealed that, among the degradation products identified by Gas Chromatography–Mass Spectrometry method (GC-MS) during the early stages of 2-naphthol degradation, the formation of 1,2-naphthalenedione (1,2-naphthoquinone) correlated with the observed increase in toxicity toward *Daphnia magna* compared with the parent compound [26]. With longer plasma treatment toxicity significantly decreased. These findings highlight the importance of including toxicity assessments in dye degradation studies.

Biological toxicity assessment methods are gaining significance for their ability to comprehensively evaluate the environmental impacts of wastewater, particularly by capturing synergistic and antagonistic effects among toxic substances—interactions often missed by conventional chemical analyses [27,28]. Among bioassays, *Lepidium sativum* (garden cress) is a widely utilized model organism in phytotoxicity studies due to its rapid germination, high sensitivity to a wide range of pollutants, and its capacity to detect subtle environmental changes, especially through root growth inhibition [29,30]. The literature underscores its broad application in evaluating the phytotoxicity of various contaminants, including individual compounds like parabens [31] and complex mixtures such as dye-containing wastewater [30,32]. For instance, Gizaw and Habtu (2022) demonstrated that catalytic wet oxidation effectively reduced the toxicity of Reactive Red 2 dye toward *Lepidium sativum* [30], while Hashem et al. (2022) highlighted the potential of *Lepidium sativum* seed powder as a biosorbent for removing Acid Orange 142 dye [32].

The removal of dyes, as well as harmful degradation products derived from dyes, can be particularly challenging due to their solubility and resistance to conventional biological treatment methods [33]. Consequently, various advanced treatment approaches have been explored, including advanced oxidation processes (AOPs) such as ozonation, Fenton and photo-Fenton reactions, and UV/H_2_O_2_, which are capable of degrading dyes, aniline or other dye-originated pollutants into less toxic intermediate compounds [29,30,31,34]. Adsorption techniques employing activated carbon or modified clays, along with membrane filtration, have also shown promising results [30,31,32,34]. However, achieving complete COD reduction remains difficult [34], and many of these methods carry the risk of generating additional toxic by-products. This highlights the necessity for integrated treatment systems and continuous monitoring to ensure effective and safe removal of both dyes and their degradation products [27,28].

In the presented study, an attempt was made to investigate the phytotoxicity of the textile dye Reactive Black 5 (RB5) in both aqueous solutions and wastewater, as well as the harmful effects of its degradation by-products—specifically an aniline derivative (4-chloroaniline) and a naphthol compound (H-acid). The primary objective was to assess the phytotoxic effects before and after ozonation. The study was based on the hypothesis that enhanced degradation of textile contaminants during catalytic ozonation would be accompanied by a reduction in phytotoxicity, although the biological response may also depend on the formation of transformation products and the complexity of the wastewater matrix. Particular attention was given to evaluating the influence of process intensification through the use of various catalysts, including Al_2_O_3_ (obtained by thermal treatment of kantal structured substrate) and plasma-deposited metal compounds (based on Co_3_O_4_ and Fe_2_O_3_ as well as W-compounds), on the reduction in phytotoxicity. Furthermore, the authors aimed to correlate the observed phytotoxic effects with the degree of degradation of the tested substances, considered together with changes in the COD indicator. The novelty of the study lies in linking the efficiency of ozone-based treatment with the resulting phytotoxic response and extending this assessment from model dye solutions and selected transformation products to complex real textile wastewater. The test organism used was garden cress (*Lepidium sativum*), an agriculturally relevant plant species with potential application as a bioindicator for assessing the feasibility of extending wastewater reuse boundaries in agricultural irrigation.

## 2. Results

The study consisted of a two-stage investigation aimed at evaluating the efficiency and environmental impact of advanced oxidation process applied to dye-containing wastewater. The first stage focused on the application of ozonation and catalytic ozonation, with particular attention given to a group of heterogeneous catalysts deposited on structured supports. The objective was to assess the catalytic enhancement of pollutant degradation, quantified by the percentage reduction in absorbance at the characteristic absorption maximum (λmax), and the reduction in organic load, evaluated using chemical oxygen demand (COD). In the second stage, the impact of both ozonation and catalytic ozonation on the phytotoxicity of dye solutions, raw wastewater, and intermediate degradation products was examined. These analyses provided new and relevant data on the use of *Lepidium sativum* as a sensitive bioindicator, contributing to a deeper understanding of the ecological safety of treated effluents.

### 2.1. Catalytic Ozonation

The presented study demonstrates the application of catalytic ozonation across a diverse set of samples, including model solutions containing Reactive Black 5 (RB5) dye, 4-chloroaniline, and H-acid (5-amino-4-hydroxynaphthalene-2,7-disulfonic acid), as well as real wastewater samples (characterized in Section 4.1). All experiments were conducted under uniform conditions, employing a consistent ozone dosage to enable a reliable comparison of degradation efficiency across different pollutant matrices. The study further investigates the effect of process intensification through the use of various catalysts—obtained by the plasma-deposition method, based on metal oxides (Co_3_O_4_ and Fe_2_O_3_) as well as W-compounds and Al_2_O_3_ obtained by thermal treatment of kantal knitted gauze which serve also as structured support [35,36,37].

**Figure 1:**
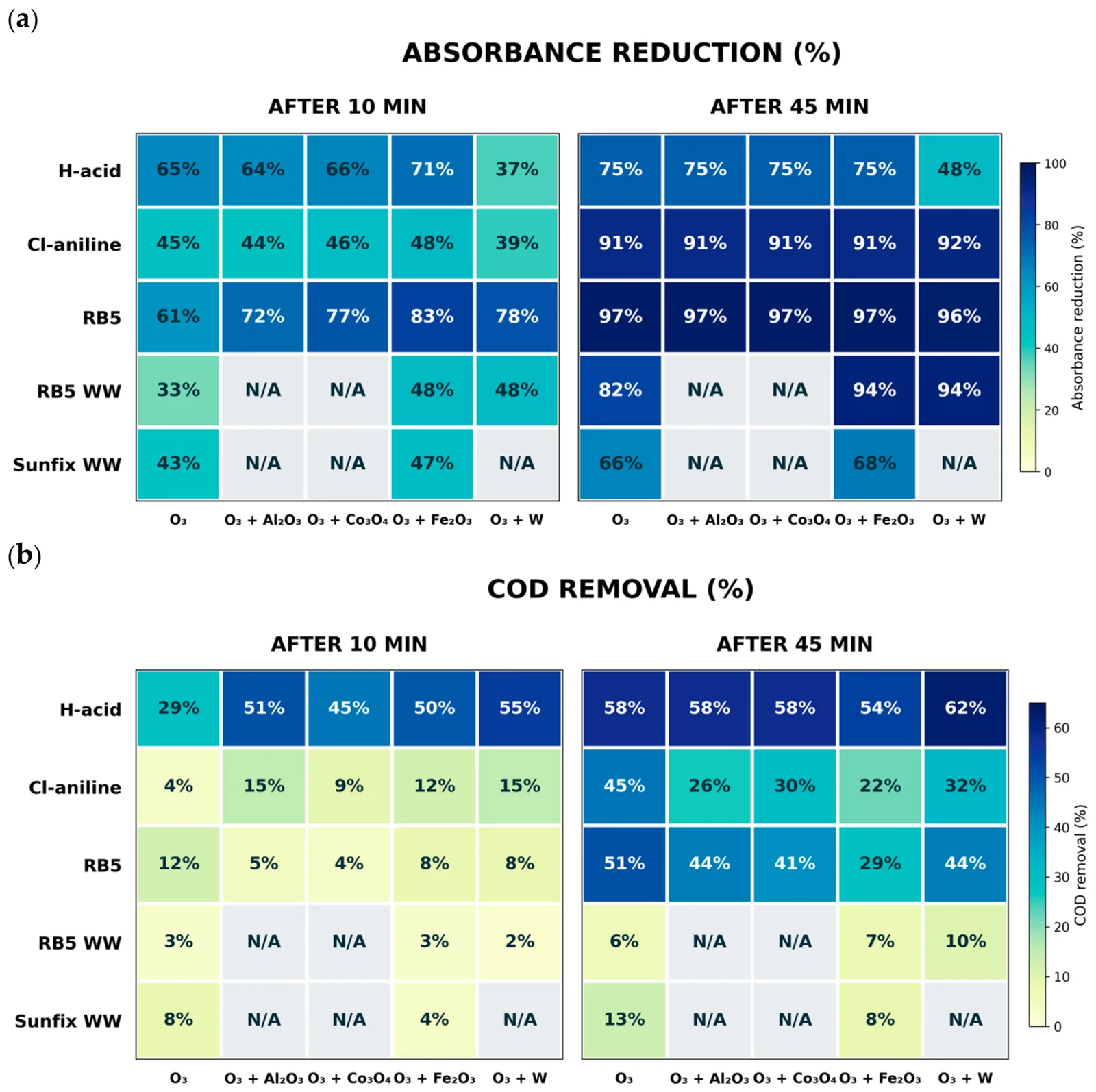
Comparison of (**a**) absorbance reduction (%) and (**b**) chemical oxygen demand (COD) removal (%) after 10 and 45 min of conventional and catalytic ozonation of model pollutants and industrial textile wastewater (pH ≈ 10). Absorbance reduction data are presented here for the first time, based on experimental results partly reported in [35] for Al_2_O_3_-, Co_3_O_4_-, and Fe_2_O_3_-based catalysts and in [37] for W-based catalysts. Data for Sunfix WW were obtained in the present study [this work].

The data presented in Figure 1 illustrate the effectiveness of conventional and catalytic ozonation across different pollutant matrices, evaluated in terms of absorbance and COD reduction. Absorbance reduction, determined at the λmax, was used to assess pollutant transformation and, for dye-containing samples, decolorization. Both parameters were evaluated after 10 and 45 min of treatment to compare the initial and final process efficiencies. A comprehensive summary of the numerical absorbance and COD reduction data is provided in Table S2 (Supplementary Materials).

Absorbance reduction (Figure 1a) varied with pollutant type and catalytic system. For the RB5 model solution, catalytic ozonation enhanced the initial absorbance reduction from 61% (O_3_) to 72–83% after 10 min, with Fe_2_O_3_ exhibiting the highest efficiency. After 45 min, all systems achieved 96–97% reduction. Cl-aniline showed a similar final response, reaching 91–92% absorbance reduction, compared with 39–48% after 10 min. For H-acid, absorbance reduction after 45 min reached 75% for O_3_, Al_2_O_3_, Co_3_O_4_, and Fe_2_O_3_, whereas the W-based system achieved 48%. In industrial wastewater, the final absorbance reduction reached 82% for RB5 wastewater (WW) treated with O_3_ and 94% with Fe_2_O_3_ or W-based catalysts. For Sunfix WW, the corresponding values were 66% and 68% for O_3_ and Fe_2_O_3_, respectively.

COD reduction (Figure 1b) exhibited a different dependence on pollutant type and catalyst. For H-acid, COD removal after 10 min increased from 29% with O_3_ to 45–55% in catalytic systems, reaching 54–62% after 45 min, compared with 58% for O_3_. The highest final COD reduction was obtained with the W-based catalyst (62%). For Cl-aniline, catalytic systems achieved 9–15% COD removal after 10 min, compared with 4% for O_3_. However, after 45 min, conventional ozonation provided the highest COD removal (45%), while catalytic systems achieved 22–32%. For RB5, COD removal after 45 min reached 51% with O_3_ and 29–44% with catalysts, despite nearly complete absorbance reduction. In industrial wastewater, COD removal remained considerably lower, reaching 6–10% for RB5 WW and 8–13% for Sunfix WW after 45 min.

Overall, absorbance reduction generally exceeded COD removal, particularly for dye-containing matrices. The greatest differences were observed for RB5 WW, where 82–94% absorbance reduction corresponded to only 6–10% COD removal. Conversely, H-acid treated with the W-based catalyst exhibited higher COD removal (62%) than absorbance reduction (48%). These results establish the quantitative differences between spectrophotometric and COD responses across the investigated pollutants and catalytic systems.

### 2.2. Phytotoxicity of Reactive Black 5 and Dye-Containing Textile Wastewater

The data presented in Table 1 provide a comparative overview of the phytotoxic effects of simulated and real textile wastewater samples on the germination and early growth of *Lepidium sativum* seeds. Key parameters analyzed include the number of germinated seeds, average root length, Relative Seed Germination (RSG), Relative Root Growth (RRG), and the Germination Index (GI), all of which serve as indicators of plant response to potential toxicants. The experimental setup included a control sample (clean water) and four test samples: wastewater containing Sunfix Yellow, Red, and Blue dyes (Sunfix WW); wastewater with Reactive Black 5 (RB5 WW); and a pure RB5 dye solution. These results enable a detailed evaluation of the phytotoxicity associated with both complex dye mixtures and individual dye compounds.

In phytotoxicity assessments, the aqueous solution of RB5 supported full seed germination (Relative Seed Germination, RSG = 100%); however, root elongation was significantly inhibited, with RRG reduced to 50%, yielding a GI of 50%. This indicates a moderate phytotoxic effect attributable primarily to impaired root development rather than inhibition of germination itself. Although the initial GI was 50%, catalytic ozonation resulted in a substantial improvement. After 45 min of treatment, GI increased to 65%, accompanied by a near-restoration of root development.

In contrast, textile wastewater samples derived from dyebaths containing either Sunfix (a trichromatic dye mixture) or RB5 completely suppressed both germination and root growth, with GI, RSG, and RRG values remaining at 0%. Notably, ozonation and catalytic ozonation processes failed to improve the phytotoxic profile of these wastewater samples, as no recovery in plant response was observed post-treatment.

The lack of detoxification efficacy in these samples may be associated with the complex composition of the textile effluent matrix, potentially combined with the effect of elevated pH (>10). Although alkalinity may affect seed germination and root development, Bonanomi et al. (2006) [38] observed a decrease in *Lepidium sativum* root length at pH 8.8 compared with the control; however, according to their statistical analysis, this difference was not significant. Similarly, Bożym et al. (2021) [39] indicated that pH may contribute to the phytotoxic response of *Lepidium sativum* in complex matrices, where its effect can occur in combination with other physicochemical properties and contaminants. Therefore, the response observed in the present study is more likely to reflect the combined effects of high alkalinity and other constituents of the treated effluent rather than the effect of pH alone. The extreme phytotoxicity is likely the result of synergistic interactions among multiple components present in the wastewater, including dyes, salts, surfactants, and alkaline agents. For example, a salt concentration above 100 mg/L alone results in significantly reduced plant growth [40,41]. Therefore, the very high NaCl concentration in the RB5 WW (~75 g/L) can be considered a major contributor to salt-induced osmotic stress, potentially explaining the complete inhibition of seed germination observed for this sample.

These findings emphasize that, although the isolated dye RB5 exhibits only moderate phytotoxicity, its formulation within real textile wastewater significantly amplifies its ecological impact, highlighting the necessity for more tailored and matrix-specific treatment approaches.

### 2.3. Phytotoxicity of By-Products

**Figure 2:**
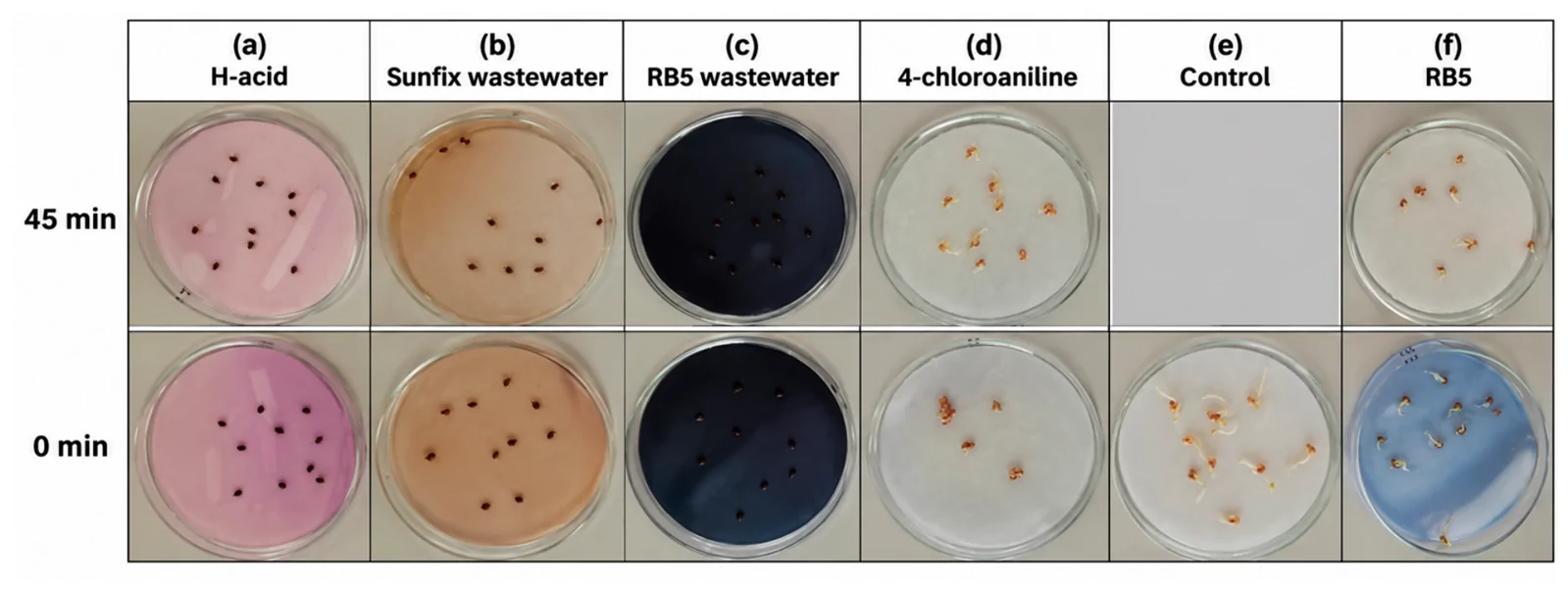
Representative photographs of Lepidium sativum seed germination and root development in (**a**) H-acid, (**b**) Sunfix WW, (**c**) RB5 WW, (**d**) 4-chloroaniline, (**e**) control, and (**f**) RB5 solution, before treatment (0 min, **bottom row**) and after 45 min of single ozonation (**top row**). Photo by the authors.

In the subsequent phase of the study, attention was directed toward the potential phytotoxicity of by-products formed during the ozonation process. The investigated transformation products were selected based on their previous identification during RB5 ozonation in a directly related study and on degradation pathways established in our earlier research [24,35]. Thus, the selection of these specific compounds was directly based on our previous experimental studies of RB5 ozonation and its transformation pathways. These transformation products can, in some cases, exhibit greater toxicity than the parent compounds, thereby limiting the overall effectiveness of the treatment. To assess this risk, phytotoxicity was evaluated at different stages of ozonation. This progressive improvement in GI over time is visually supported by Figure 2, which illustrates enhanced seedling vigor and root elongation following prolonged ozonation, suggesting a gradual decrease in phytotoxic effects as oxidation proceeds.

Table 2 presents the effects of different treatment methods on the germination parameters of *Lepidium sativum* seeds exposed to chloroaniline and H-acid by-products at varying ozonation durations (0, 10, and 45 min).

For chloroaniline, the number of germinated seeds was consistently lower than the control (10 seeds) across all treatments. Single ozonation (O_3_) maintained 7 germinated seeds at 0 and 10 min, dropping to 6 at 45 min. Catalytic systems using Al_2_O_3_, Co_3_O_4_, Fe_2_O_3_, and W-based catalysts followed a similar trend, generally maintaining 7 germinated seeds at 0 min and slightly declining to 6 at 45 min—except for Co_3_O_4_, which maintained full germination throughout the treatment duration. Given the overall high phytotoxicity of chloroaniline and its oxidation products, this stable germination rate is a relatively positive outcome.

In contrast, average root length dropped sharply from 2.6 cm in the control to 0.4 cm with single ozonation at 0 and 10 min, and further to 0.3 cm at 45 min. All catalysts followed similar patterns, with minor time-dependent decreases in root length, reaching a minimum of 0.2 cm after 45 min. Relative Seed Germination (RSG) remained at 70% for most conditions at 0 and 10 min, decreasing to 60% at 45 min. However, Relative Root Growth (RRG) exhibited a substantial decline, starting at 15.4% and decreasing to as low as 7.7% for Co_3_O_4_, Fe_2_O_3_, and W-based catalysts after 45 min. Consequently, the Germination Index (GI) decreased from 10.8% at 0 and 10 min to a minimum of 4.6% at 45 min under catalytic conditions. These results indicate persistent phytotoxic effects that were not mitigated by catalytic ozonation.

For H-acid, seed germination was also impaired relative to the control. The number of germinated seeds decreased from 10 (control) to 6 with single ozonation at 0 and 10 min, and further declined to 5 at 45 min. Catalytic systems produced similar results, with Al_2_O_3_ and Co_3_O_4_ reducing germination to 5 seeds at 45 min. Average root length exhibited a sharp decline from 2.6 cm in the control to 0.1 cm at 0 min, with further reductions to 0.08 cm at 10 and 45 min across most treatments. The lowest values (0.07 cm) were observed for Co_3_O_4_, Fe_2_O_3_, and W-based catalysts at the final time point.

RSG remained stable at 60% for most treatments at early stages but dropped to 50% for certain catalysts at 45 min. RRG was drastically reduced, starting at 3.8% and falling as low as 2.3% for Fe_2_O_3_ at 45 min. Correspondingly, GI declined from 2.3% at 0 min to 1.5% at 45 min with single ozonation. The lowest GI (1.2%) was observed for Fe_2_O_3_ at the final time point, indicating that catalytic treatments did not substantially alleviate toxicity.

The data indicate that both chloroaniline and H-acid by-products severely impair seed germination and root elongation. None of the tested treatment strategies—including single ozonation and catalytic ozonation with Al_2_O_3_, Co_3_O_4_, Fe_2_O_3_, or W-based materials—substantially improved phytotoxicity outcomes. In fact, root growth remained strongly inhibited across all conditions, and GI values declined progressively with increasing treatment duration. These findings underscore the persistent toxic nature of oxidation by-products and suggest that extended or more selective treatment approaches may be required to effectively reduce their ecological risk. The results also highlight that the degradation of these compounds must proceed to a more advanced stage, as the intermediates present at the tested points of the process remain clearly harmful. This emphasizes both the relevance of the current research and the necessity for continued, in-depth investigation to develop more effective detoxification strategies.

## 3. Discussion

The phytotoxicity assessment using *Lepidium sativum* revealed distinct differences in the biological impact of various textile wastewater components and their degradation products during catalytic ozonation. The GI, which integrates seed germination and root elongation, was reliable for evaluating detoxification efficiency.

### 3.1. RB5 Dye and Real Textile Wastewater: Moderate Toxicity and Effective Detoxification

Although the initial GI value of the RB5 solution was 50%, indicating moderate phytotoxicity associated with inhibited root elongation (RRG = 50%), catalytic ozonation resulted in partial detoxification. After 45 min, absorbance reduction reached 96–97%, while COD removal ranged from 29% to 51%, depending on the treatment system. For the W-based catalyst, absorbance and COD reductions were 96% and 44%, respectively. These results indicate extensive degradation of the dye chromophore accompanied by a less pronounced reduction in the chemically oxidizable organic load.

After 45 min of treatment, GI increased to approximately 65%, indicating a partial recovery of root growth rather than complete elimination of phytotoxicity. This improvement was accompanied by substantial absorbance reduction, suggesting that transformation of the dye structure contributed to the observed biological response. However, the persistence of residual COD indicates that organic constituents, including potential oxidation intermediates, remained in the treated solution. These findings are consistent with previous literature reports, where azo dye degradation products can exhibit lower toxicity than the parent dye molecule when properly mineralized [3,9]. Indeed, the partial detoxification observed suggests effective cleavage of chromophoric structures and oxidation of aromatic amines, which are known to interfere with plant metabolic pathways [27].

In contrast, untreated industrial wastewater samples containing RB5 or Sunfix dyes completely inhibited seed germination (GI = 0%). After 45 min of treatment, RB5 WW exhibited 82–94% absorbance reduction but only 6–10% COD removal, whereas Sunfix WW showed 66–68% absorbance reduction and 8–13% COD removal. These results demonstrate a substantial discrepancy between decolorization and the reduction in the overall chemically oxidizable organic load in complex wastewater matrices. Since phytotoxicity was not assessed after treatment of these industrial wastewaters, their detoxification cannot be established from the physicochemical results alone. The complete inhibition of germination in untreated wastewater may be associated with matrix constituents, particularly high salinity, as well as other dissolved substances and alkaline pH. Similar observations concerning the phytotoxicity of textile dye mixtures and wastewater constituents have been reported in the literature [32,42].

### 3.2. Persistent Toxicity of 4-Chloroaniline and H-Acid

A particularly notable outcome was the severe inhibition caused by 4-chloroaniline and H-acid, with initial GI values of 10.8% and 2.3%, respectively, which decreased after 45 min of treatment (GI = 4.6% and 1.2%, respectively, using Fe_2_O_3_). Despite their high resistance to oxidation, absorbance reductions of 91–92% for 4-chloroaniline and 48–75% for H-acid were achieved after 45 min, accompanied by COD reductions of 22–45% and 54–62%, respectively. These reductions can be considered substantial, particularly for H-acid, although they did not correspond to improved biological responses. However, the continued and even intensified phytotoxicity indicates that residual parent compounds and/or transformation products generated under the applied conditions may retain biological activity.

These compounds are known for their high toxicity and poor biodegradability, which limits the efficiency of oxidation processes under mild conditions [30]. The observed absorbance and COD reductions confirm substantial changes in the physicochemical characteristics of the treated solutions, but these changes were insufficient to eliminate phytotoxic effects entirely. Previous work has indicated that chloroaniline derivatives are highly resistant to advanced oxidation and may require additional steps (e.g., increased ozone dosage or pH adjustment) for significant detoxification [29]. The limited response in GI confirms their persistent phytotoxic nature and further emphasizes the need to optimize catalytic ozonation parameters.

### 3.3. Lepidium sativum as a Sensitive Bioindicator

The results validate using *Lepidium sativum* as a sensitive and practical bioindicator for assessing wastewater toxicity. In contrast to microbial assays (e.g., *Vibrio fischeri*), *Lepidium sativum* offers insight into the effects of toxicants on multicellular eukaryotes, especially regarding growth regulation at the cellular level [27,28]. Its rapid germination and clear morphological responses (e.g., root elongation inhibition) enable fast and reproducible evaluation of detoxification processes. Notably, *Lepidium sativum* was more sensitive to residual toxicity in the RB5 and chloroaniline solutions than microbial endpoints reported in our earlier work, highlighting its relevance in ecotoxicological assessment.

**Figure 3:**
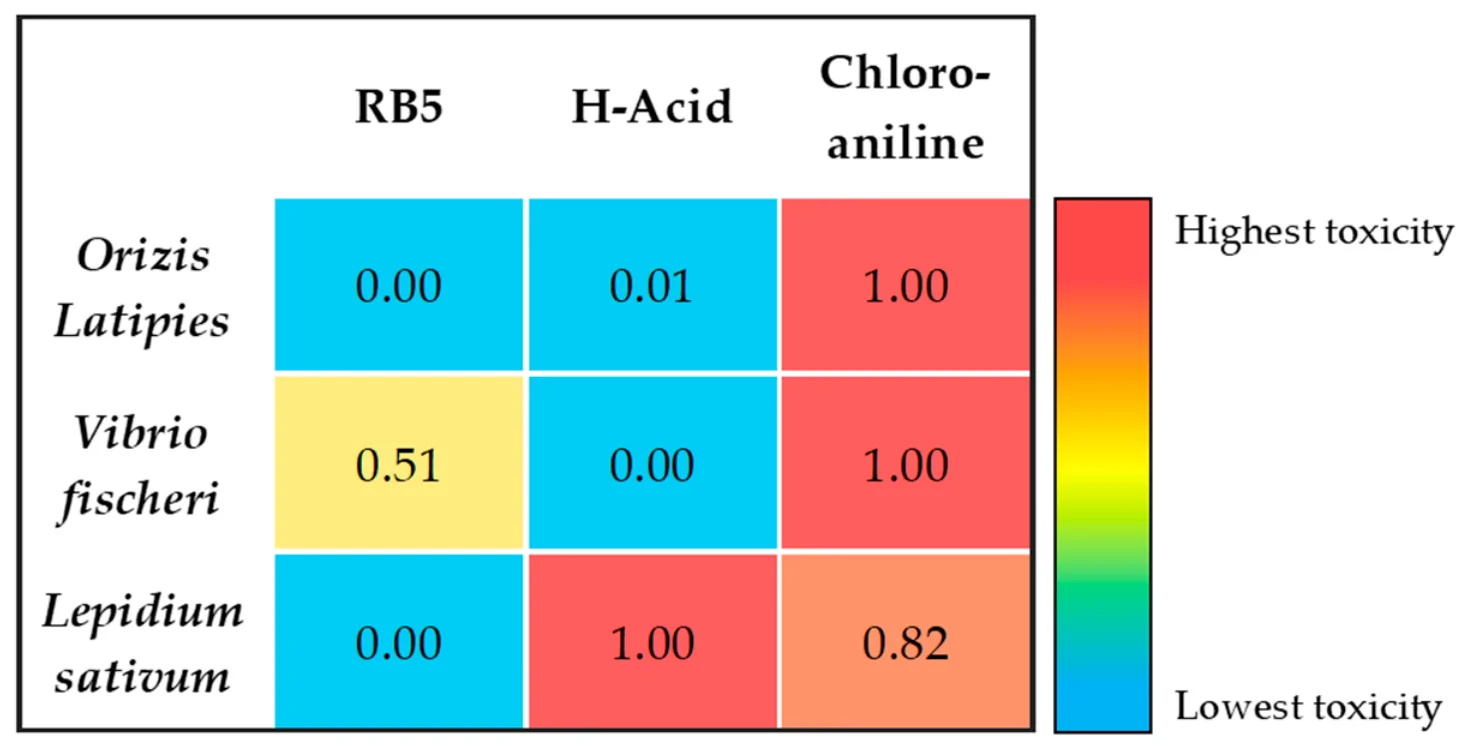
Comparative ecotoxicity profile of Reactive Black 5 (RB5), H-Acid and p-chloroaniline based on fish toxicity (TU derived from LC50) from QSAR Toolkit database, bacterial toxicity (TU derived from EC50 for *Vibrio fischeri*) from [35] and phytotoxicity toward *Lepidium sativum* from present study. Values were normalized within each endpoint to facilitate comparison of relative toxicity among the tested compounds.

To further assess the sensitivity of *Lepidium sativum* as a bioindicator, its response was compared with those of *Vibrio fischeri* and *Oryzias latipes*, representing different taxonomic groups and biological levels of organization. This comparison provides a broader ecotoxicological perspective and highlights species-specific differences in sensitivity to the investigated contaminants. A comparison of the toxicological results obtained for *Vibrio fischeri* and the available toxicological data for *Oryzias latipes* retrieved from the QSAR Toolbox database (version 4.9) is presented in Figure 3. The original data are provided in Table S3 and calculations explained in Text S3 in the Supplementary Materials. The results indicate that the selected degradation products of RB5, namely p-chloroaniline and H-acid, exhibit higher toxicity than the parent compound. A particularly noteworthy observation is the sensitivity of *Lepidium sativum*, which responded adversely to both compounds. In contrast, *Oryzias latipes* showed lower relative sensitivity to H-acid, highlighting differences in species-specific toxicological responses.

*Lepidium sativum* showed a clear response to RB5 exposure, as evidenced by a reduction in the growth index to 50% of the control. However, the degradation products H-acid and p-chloroaniline exerted substantially stronger phytotoxic effects, reducing the growth index to 2.3% and 10.8%, respectively. These results suggest that the transformation of RB5 may lead to the formation of products with enhanced phytotoxicity. Therefore, effective wastewater treatment should ensure not only the removal of parent dyes but also the elimination of potentially more toxic transformation products, as dye degradation alone does not necessarily imply detoxification.

### 3.4. Catalyst Stability and Potential Contribution of Metal Leaching

Potential metal leaching from the catalysts was considered as an additional factor that could affect the phytotoxicity of the treated samples. Catalyst stability and metal release were investigated in detail in our previous studies directly related to the catalytic ozonation systems applied here. No detectable metal release into the treated water was observed under the investigated conditions, particularly at pH 10 [35]. The stability of the tungsten-based catalyst was additionally investigated in our earlier work [37]. Although this catalyst exhibited lower stability, the concentration of tungsten in the treated water remained below the analytical detection limit. Based on these previous findings, metal leaching is not expected to represent a major factor contributing to the phytotoxic effects observed in the present study.

## 4. Materials and Methods

### 4.1. Chemicals and Wastewater

The experiments used model solutions containing RB5 dye, 4-chloroaniline, and H-acid (5-amino-4-hydroxynaphthalene-2,7-disulfonic acid). All chemicals were analytical grade and supplied by Boruta-Zachem (Bydgoszcz, Poland). The solutions were prepared by dissolving substances in distilled water, resulting in concentrations of 50 mg/L for RB5 dye and 10 mg/L for the by-products (4-chloroaniline and H-acid) with pH 10 phosphate buffer (sodium dihydrogen phosphate NaH_2_PO_4_, and sodium hydroxide NaOH, both analytical grade, and purchased from Chempur (Piekary Śląskie, Poland)). The industrial wastewater originated from dyeing of cotton fabric was used in experiments. The dye and the auxiliaries of technical grade were present in this wastewater: Remazol Ultraschwarz NN (industrial product based on RB5, DyStar Global Holdings Pte. Ltd., (Singapore, Singapore)), Sunfix Yellow SPD conc., Sunfix Red SPD conc., Sunfix Blue SPD conc. industrial products by OHYOUNG Industrial Co., Ltd., (Siheung-si, Gyeonggi-do, Republic of Korea), industrial dyeing assistant—Perigen LDR (SAA—naphthalenesulfonic acid and carboxylates’ mixture, Textilchemie Dr. Petry Co. (Reutlingen, Germany)) as well as NaCl by Inowrocławskie Kopalnie Soli SOLINO S.A. (Inowrocław, Poland), NaOH, and Na_2_CO_3_ by JARBUR CHEMICALS (Szydłowiec, Poland). The wastewater was taken from an equalization reservoir (average wastewater from several dyeing operations). The characteristic of the wastewater was presented in Table 3.

### 4.2. Catalyst Preparation

The thin-film catalysts were prepared using the plasma-enhanced chemical vapor deposition (PECVD) method. An RF (13.56 MHz) parallel-plate reactor was employed [10]. The catalyst support was structured packing in the form of three-dimensional knitted gauzes made of Kanthal HS wire (FeCrAl alloy, 0.11 mm in diameter) supplied by TermTech (Marki, Poland). Before catalyst deposition, the gauzes were pre-coated with an aluminum oxide (Al_2_O_3_) layer through thermal treatment in air at 900 °C for 48 h [35,36,37]. Detailed characterization and images of the packing structure are available in reference [35,37]. The deposition procedure of the thin-film catalysts based on metal oxides (Co_3_O_4_ and Fe_2_O_3_) and tungsten-based materials together with characteristics of prepared catalyst materials are described in detail in reference [35,36,37].

### 4.3. Catalytic Test

The semi-batch glass bubble column (31 cm high and 7 cm in diameter) with a stationary liquid phase of 1 L volume supported the catalytic experiment (heterogeneous catalytic ozonation, HCO). The reactor was equipped in baffles of selected geometry (enabling the ozone flow), which were carriers for the catalyst. The precise description given under the patent claim P.445362 (European analog application EP23215146.4). Ozone gas produced by the BMT 802N generator (BMT Messtechnik GmbH, Stahnsdorf, Germany) from cylinder compressed oxygen (O_2_ purity 99.5%) by Linde Gas (Kraków, Poland) was introduced into the system through a G3 ceramic frit placed in the reactor bottom. The gas-phase ozone concentration at the reactor inlet and outlet was monitored through the BMT 963 measurer (BMT Messtechnik GmbH, Stahnsdorf, Germany). The detailed procedure with the applied ozone dose of 0.015 g O_3_/L (15 mg O_3_/L)was described elsewhere [35].

### 4.4. Analysis

The color was determined by spectrophotometer Genesys 180 by Thermo Fisher Scientific (Waltham, MA, USA), through a calibration plot based on Lambert-Beer’s law conversion into concentration. *Absorbance removal* (Abs removal, %) was calculated according to Equation (1):

(1) $$Absorbance\;removal\;(\%)=\frac{A_{0}-A_{t}}{A_{0}}\cdot 100\%$$

where *A_0_* and *A_t_* are the initial absorbance and absorbance measured after treatment time (*t*), respectively. Measurements were performed at the characteristic absorption maxima (λ_max_): 596 nm for RB5 solution and RB5 wastewater, 604 nm for Sunfix WW (purple-like coloration), 376 nm for H-acid, and 380 nm for 4-chloroaniline.

Chemical oxygen demand (COD) was determined using LCK cuvette tests (HACH, Loveland, CO, USA) and a DR 3900 spectrophotometer (HACH, Loveland, CO, USA), following the manufacturer’s instructions. *COD removal efficiency* was calculated according to Equation (2):

(2) $$COD\;removal\;(\%)=\frac{{COD}_{0}-{COD}_{t}}{{COD}_{0}}\cdot 100\%$$

where *COD*_0_ and *COD_t_* represent the initial COD and COD measured after treatment time *t*, respectively, expressed in mg O_2_ L^−1^.

The residual dissolved ozone was routinely monitored after treatment by direct spectrophotometric measurement at 260 nm (ε ≈ 3000 M^−1^ cm^−1^) and was consistently below the detection limit in the ozonated samples.

The toxicity of the tested solutions was evaluated using a phytotoxicity assay with *Lepidium sativum* (garden cress) seeds from PlantiCo (Zielonki-Parcela, Poland). The experimental procedure was based on the methodology described by Walter et al. [27] incorporating standard steps and conditions for germination testing.

The tests were conducted in Petri dishes containing 10 seeds on a Munktell paper filter from Ahlstrom Munksjo (Espoo, Finland). Subsequently, 5 mL of the tested solution or distilled water (control sample) was added. The dishes were incubated for 48 h in the dark at 25 °C and a relative humidity of approximately 45%.

Toxicity toward *Lepidium sativum* was evaluated using the Germination Index (GI), which combines the effects of Relative Seed Germination (RSG) and Relative Root Growth (RRG) into a single phytotoxicity indicator. These parameters were calculated according to the following formulas:

(3) $$RSG\;(\%)=\frac{Number\;of\;seeds\;germinated\;in\;treatment}{Number\;of\;seeds\;germinated\;in\;control}\times 100\%$$

(4) $$RRG\;(\%)=\frac{Average\;root\;length\;in\;treatment\;}{Average\;root\;length\;in\;control}\times 100\%$$

(5) $$GI\;(\%)=\frac{RSG\times RRG}{10}$$

The analysis was performed for samples taken at 0, 10, and 45 min of the catalytic ozonation process. The results were compared to the control, for which RSG and RRG values were assumed to be 100%, and the average root length was 2.6 cm. Seedling growth dimensions were measured using vernier caliper with a measurement resolution of 0.2 mm. The experimental unit was a single Petri dish containing ten Lepidium sativum seeds exposed to the same tested sample. Root length was measured individually for each seedling and is reported as the mean ± standard deviation (SD) of these measurements. This approach comprehensively evaluated the tested solutions’ impact on *Lepidium sativum* development.

## 5. Conclusions

The present study confirms that *Lepidium sativum* is a sensitive and effective bioindicator for evaluating the phytotoxicity of textile wastewater and its constituents. The GI provided a robust and biologically relevant measure of detoxification efficiency during catalytic ozonation, enabling the differentiation between moderate toxicity, as observed for RB5, and high toxicity, as seen in 4-chloroaniline, H-acid, and dye mixtures present in textile wastewater.

Catalytic ozonation resulted in partial detoxification of RB5, as indicated by an increase in the GI from approximately 50% to 65% after 45 min of treatment; however, the available phytotoxicity data do not allow the individual contributions of the investigated catalysts to be distinguished, but rather a process generally. This improvement was consistent with the observed decrease in COD, which indicated substantial degradation of the dye’s organic load. In contrast, despite achieving moderate to satisfactory COD reduction for recalcitrant compounds such as 4-chloroaniline and H-acid (COD_45_/COD_0_ as low as 0.38 for H-acid), phytotoxicity remained pronounced or even increased, suggesting that intermediate oxidation products retained high biological activity. Similarly, textile wastewater containing dye mixtures demonstrated complete inhibition of seed germination and no GI recovery, regardless of treatment time, despite partial COD removal.

These findings emphasize the importance of integrating biological toxicity testing into the evaluation of AOPs. While COD serves as a valuable indicator of chemical degradation, it does not necessarily correlate with detoxification, particularly in complex or halogenated matrices. Therefore, while catalytic ozonation holds promise as a treatment strategy, its efficacy is strongly influenced by the structural properties of the pollutants. Process optimization and the incorporation of complementary post-treatment steps may be essential to ensure complete detoxification and minimize ecological risk in the treatment of persistent industrial contaminants.

## 6. Patents

The construction of the bubble column and catalyst usage are under the Polish patent claim application (P.445362) and European consolidated patent claim (analogs application EP23215146.4).

## Figures and Tables

**Table 1:** Germination parameters for the control sample, Reactive Black 5 and Dye-Containing Textile Wastewater before treatment.

Parameter	Sample Type			
	Clean Water (Control)	Sunfix WW	RB5 WW	RB5 Solution
Number of germinated seeds	10	0	0	10
Average root length, cm	2.60 ± 0.08	0	0	1.30 ± 0.06
RSG, %	100	0	0	100
RRG, %	100	0	0	50
GI, %	100	0	0	50

**Table 2:** Germination parameters for the control sample and by-products (chloroaniline and H-acid) before and after treatment (10 and 45 min).

Parameter	Chloroaniline															
	Control	Single O_3_			Al_2_O_3_			Co_3_O_4_			Fe_2_O_3_			W-Based		
Time, min	-	0	10	45	0	10	45	0	10	45	0	10	45	0	10	45
**Number of germinated seeds**	10	**7**	7	6	**7**	7	6	**7**	7	7	**7**	7	6	**7**	6	6
**Average root length, cm**	2.60 ± 0.08	0.40 ± 0.10	0.40 ± 0.09	0.30 ± 0.09	0.40 ± 0.10	0.30 ± 0.08	0.30 ± 0.09	0.40 ± 0.10	0.30 ± 0.09	0.20 ± 0.06	0.40 ± 0.10	0.30 ± 0.07	0.20 ± 0.07	0.40 ± 0.10	0.20 ± 0.07	0.20 ± 0.07
**RSG, %**	100	70	70	60	70	70	60	70	70	70	70	70	60	70	60	60
**RRG, %**	100	15.4	15.4	11.5	15.4	11.5	11.5	15.4	11.5	7.7	15.4	11.5	7.7	15.4	7.7	7.7
**GI, %**	100	10.8	10.8	6.9	10.8	8.1	6.9	10.8	8.1	5.4	10.8	8.1	4.6	10.8	4.6	4.6
**Parameter**	**H-acid**															
	**Control**	**Single O_3_**			**Al_2_O_3_**			**Co_3_O_4_**			**Fe_2_O_3_**			**W-based**		
**Time, min**	-	0	10	45	0	10	45	0	10	45	0	10	45	0	10	45
**Number of germinated seeds**	10	6	6	5	6	6	5	6	6	6	6	5	5	6	6	5
**Average root length, cm**	2.60 ± 0.08	0.10 ± 0.04	0.08 ± 0.02	0.08 ± 0.03	0.10 ± 0.04	0.08 ± 0.02	0.08 ± 0.03	0.10 ± 0.04	0.07 ± 0.02	0.07 ± 0.02	0.10 ± 0.04	0.80 ± 0.03	0.60 ± 0.02	0.10 ± 0.04	0.07 ± 0.02	0.07 ± 0.02
**RSG, %**	100	60	60	50	60	60	50	60	60	60	60	50	50	60	60	50
**RRG, %**	100	3.8	3.1	3.1	3.8	3.1	3.1	3.8	2.7	2.7	3.8	3.1	2.3	3.8	2.7	2.7
**GI, %**	100	2.3	1.8	1.5	2.3	1.8	1.5	2.3	1.6	1.6	2.3	1.5	1.2	2.3	1.6	1.3

**Table 3:** Textile wastewater parameters.

Parameter	Wastewater RB5	Wastewater Sunfix
Dye concentration, mg/L	750.00	63.0
NaCl concentration, mg/L	75 thous.	5 thous.
pH	10.6	10.3
COD, mg/L	2820	1987
TOC, mg/L	152.3	39.4

## Data Availability

The raw data supporting the conclusions of this article will be made available by the authors on request.

## Supplementary Materials

The following supporting information can be downloaded at: https://www.mdpi.com/article/10.3390/molecules31214017/s1, Text S1, Text S2, Text S3, Table S1. Experimental roadmap of the research programme on structured catalytic ozonation of textile wastewater., Table S2. Catalytic test performance., Table S3. Comparison of LC50, EC50, GI, and calculated Toxic Unit (TU) values for RB5 and its selected degradation products.
