# Supplementary material for: Phytotoxic Effects of Textile Wastewater Contaminants on Lepidium sativum: Impact of Ozone-Based Treatment
Source: Molecules. 2026 Nov 7;31(21):4017. doi: 10.3390/molecules31214017 (PMC13765345; doi:10.3390/molecules31214017)
Supplement: Supplementary file 1 [file molecules-31-04017-s001.zip › molecules-4597337-supplementary.pdf]

Supplementary materials

Text S1. The present study forms part of the broader experimental programme conducted within the TEX-WATER-REC project (SGS, Norway Grants). The programme progressed from the design and hydrodynamic characterization of a structured catalytic ozonation reactor [1], through the development, characterization and performance assessment of metal oxide catalysts [2,3], to the evaluation of pollutant transformation, oxidation efficiency and ecotoxicity [2,3]. The present investigation extends this framework by assessing the phytotoxicity of treated wastewater using Lepidium sativum, complementing the previously established physicochemical and bacterial toxicity assessments. The overall experimental roadmap is summarized in Table S1.

Table S1. Experimental roadmap of the research programme on structured catalytic ozonation of textile wastewater.

Research stage	Experimental scope and methods	Research objective
1. Reactor engineering	Semi-batch bubble column with three mesh-filled baffles [1]; 3D-knitted Kanthal supports [1–3]	Development of a structured catalytic ozonation reactor
2. Hydrodynamic characterization	Gas holdup (εG), bubble diameter, interfacial area (a), mass transfer coefficients (kLa, kL) [1]; bare and calcined Kanthal meshes [1]	Evaluation of gas–liquid mass transfer
3. Reaction regime determination	Ozone absorption and decomposition kinetics [1]; RB5 oxidation kinetics, Hatta number (Ha) and enhancement factor (E) [1]	Identification of mass transfer and chemical reaction regimes
4. Catalyst development	PECVD deposition of CoOx and FeOx [2] and W-based thin films [3]; thermal post-treatment [2,3]	Development of structured heterogeneous catalysts
5. Catalyst characterization	SEM–EDX and XPS [2,3]; surface morphology and composition [1–3]; influence of PECVD parameters and post-treatment [3]	Establishment of structure–composition relationships
6. Catalytic performance	RB5 degradation kinetics [1–3]; pH-dependent activity (pH 2, 7, 10) [2]; W-based catalyst screening at pH 10 [3]	Evaluation of catalytic activity and operating conditions
7. Catalyst stability	Seven reuse cycles [2,3]; post-ozonation surface characterization [2,3]; W-based film degradation [3]	Assessment of catalyst durability
8. Pollutant transformation	RB5-Na and RB5-OH by HPLC [2,3]; 4-chloroaniline and H-acid degradation [2]	Investigation of dye transformation and selected by-products
9. Oxidation efficiency	COD, TOC and AOS [2]; COD analysis of real textile wastewater [3]	Assessment of organic matter oxidation
10. Industrial validation	Treatment of real textile wastewater using W-based catalysts [3]; comparison with synthetic dye solutions [3]	Verification of treatment performance in industrial matrices
11. Ecotoxicological assessment	Vibrio fischeri (Microtox); acute toxicity evaluation [2,3], the phytotoxicity of treated wastewater using Lepidium sativum [this work]	Assessment of toxicity changes following ozonation

Text T2. The relationship between absorbance reduction, COD removal, and phytotoxicity was examined to assess pollutant transformation and its environmental implications (Table S2). Absorbance reduction at the characteristic absorption maximum was considered an indirect indicator of parent compound degradation (decolorization for dyes), whereas COD removal reflected the decrease in chemically oxidizable organic matter, including oxidation intermediates.

The results demonstrated pollutant- and catalyst-dependent differences between spectrophotometric and COD responses. After 45 min, the RB5 model solution exhibited extensive decolorization (A_{45}/A_0 = 0.03–0.04), accompanied by substantially higher residual COD (COD_{45}/COD_0 = 0.49–0.71). This discrepancy was particularly pronounced in industrial wastewater, where RB5 WW exhibited A_{45}/A_0 =

0.06–0.18 and COD_{45}/COD_0 = 0.90–0.94, while Sunfix WW showed A_{45}/A_0 = 0.34 and COD_{45}/COD_0 = 0.87–0.92. In contrast, H-acid exhibited comparatively greater COD reduction (COD_{45}/COD_0 = 0.38–0.46), despite a wider range of residual absorbance (A_{45}/A_0 = 0.25–0.52). For 4-chloroaniline, a pronounced decrease in relative absorbance (A_{45}/A_0 = 0.08–0.09) was accompanied by COD_{45}/COD_0 = 0.55–0.78. The catalytic effect varied among the investigated pollutants and wastewater matrices, indicating that changes in characteristic absorption bands did not consistently correspond to proportional COD removal.

Phytotoxicity assessment using *Lepidium sativum* further demonstrated that physicochemical pollutant removal did not necessarily reduce biological effects. Untreated H-acid and Cl-aniline exhibited pronounced phytotoxicity (GI = 2.3% and 10.8%, respectively), which persisted or intensified after 45 min of ozonation (GI = 1.2–1.6% and 4.6–6.9%, respectively), despite substantial absorbance and COD reductions. Root elongation was particularly inhibited (RRG = 2.3–3.1% for H-acid and 7.7–11.5% for Cl-aniline). Untreated industrial wastewaters (RB5 WW and Sunfix WW) completely inhibited seed germination (GI = 0%), whereas the model RB5 solution exhibited moderate phytotoxicity (GI = 50%), with full germination but 50% inhibition of root elongation. These observations indicate markedly greater phytotoxicity of complex industrial matrices compared with the model dye solution.

Overall, the results demonstrate that absorbance reduction, COD removal, and phytotoxicity are complementary but non-equivalent indicators of ozonation performance**.** The persistence of phytotoxicity despite substantial pollutant transformation suggests that residual parent compounds and/or oxidation intermediates may retain biological activity, emphasizing the importance of integrating ecotoxicological assessment with physicochemical monitoring.

Table S2. Catalytic test performance.

**Pollutant**	**Catalyst type**	**pH**	**Relative absorbance,**		**COD$_0$**	**Relative COD**	
			**A_{10}/A_0**	**A_{45}/A_0**		**COD_{10}/COD_0**	**COD_{45}/COD_0**
**H-acid**	-	10	0.35	0.25	50.9	0.71	0.42
	Al_2O_3		0.36	0.25		0.49	0.42
	Co_3O_4		0.34	0.25		0.55	0.42
	Fe_2O_3		0.29	0.25		0.50	0.46
	W-based		0.63	0.52		0.45	0.38
**Cl-aniline**	-	10	0.55	0.09	34.3	0.96	0.55
	Al_2O_3		0.56	0.09		0.85	0.74
	Co_3O_4		0.54	0.09		0.91	0.70
	Fe_2O_3		0.52	0.09		0.88	0.78
	W-based		0.61	0.08		0.85	0.68
**RB5**	-	10	0.39	0.03	45	0.88	0.49
	Al_2O_3		0.28	0.03		0.95	0.56
	Co_3O_4		0.23	0.03		0.96	0.59
	Fe_2O_3		0.17	0.03		0.92	0.71
	W-based		0.22	0.04		0.92	0.56
**RB5 WW**	-	10.6	0.67	0.18	1880	0.97	0.94
	Fe_2O_3		0.52	0.06		0.97	0.93
	W-based		0.52	0.06		0.98	0.90
**Sunfix WW**	-	10.3	0.57	0.34	1987	0.92	0.87
	Fe_2O_3		0.53	0.34		0.96	0.92

Table S3. Comparison of LC50, EC50, GI, and calculated Toxic Unit (TU) values for RB5 and its selected degradation products.

	Investigated spiecies	RB5	H-Acid	Chloroaniline	Source
Mortality LC50 48h, mg/L	*Orizis Latipies*	1150*	880	28	QSARTools database *data predicted by QSARTool
TU		0,09	0,11	3,57	
EC50, 15 min, mg/L	*Vibrio fischeri*	6,82	54,05	3,7	Work [2]
TU		14,7	1,9	27,0	
GI %	*Lepidium sativum*	50	2,3	10,8	Present work

Text S3. The Toxic Unit (TU) values were calculated according to the equation reported in [2]. Subsequently, the obtained TU values were normalized using the min-max normalization equation:

$$X_{norm} = \frac{X_i - X_{min}}{X_{max} - X_{min}} \quad (1)$$

The normalized values were used to construct the toxicity heat map presented in Figure 3 of the main manuscript. Growth Index (GI) values were first transformed according to the equation

$$X_i{}' = 100 - X_i \quad (2)$$

and then normalized using Equation (1) to ensure consistency with the toxicity scale adopted in the heat map.

References

[1] Bilińska, M., Bilińska, L., Kędzierska-Sar, A., & Gmurek, M. (2024). Novel Bubble Column Design Constructed for Catalytic Ozonation – Effectiveness Assessment by Hydrodynamics and Kinetic Regime Determination. Ozone: Science & Engineering. https://doi.org/10.1080/01919512.2024.2391977 (Reference [36] in the Main Text)

[2] Bilińska, M. et al. (2025). Application of metal oxides thin-film catalysts in structured catalytic ozonation reactor for dye and by-product detoxification. Scientific Reports, 15, 18580. https://doi.org/10.1038/s41598-025-02330-2 (Reference [35] in the Main Text)

[3] Kędzierska-Sar, A. et al. (2025). Stability and Reusability of Tungsten Catalyst on Structured Support in Catalytic Ozonation of Textile Wastewater. Molecules, 30, 969. https://doi.org/10.3390/molecules30040969 (Reference [37] in the Main Text)
